# Treatment of Maxillary Second Premolar with 4 Roots

**DOI:** 10.1155/2020/8634797

**Published:** 2020-05-15

**Authors:** Z. Allahem, S. AlYami

**Affiliations:** ^1^Department of Restorative Dental Sciences, College of Dentistry, King Saud University, Saudi Arabia; ^2^Department of Restorative Dental Science, College of Dentistry, Najran University, Saudi Arabia

## Abstract

*Aim*. To report a case of maxillary second premolar with four roots and four separate foramina and the use of CBCT to help identify the root canal system configuration. *Summary*. A 45-year-old female patient with a no significant medical history was referred for treatment tooth #15. The tooth was diagnosed with symptomatic irreversible pulpitis and symptomatic apical periodontitis. After taking periapical radiographs and expected uncommon morphology, CBCT was taken to help confirm canals configuration and aid in the treatment of the case. Root canals treatment was carried without complication under dental operating microscope using a rotary system and continuous wave obturation.

## 1. Introduction

The key to successful endodontic treatment lies in understanding the complexity of the root canal system and cleaning all the roots canals [1]. Incomplete instrumentation followed by incorrect obturation is the most common reason for the failure of endodontic treatment [2]. The most common morphology of the maxillary second premolar is a single root (67%), followed by two roots (30%), while the incidence rate of three roots is only 3%. An incidence of 0.3 to 2% has been seen in laboratory studies for maxillary second premolars with three separate roots [3, 4]. A study done in 1974 in the North of America reported that 75% of teeth exhibiting with one canal at the apex with type I, II, and III Vertucci classification in 48%, 22%, and 5%, respectively [3]. A recent study done by CBCT reported that type I, II, III, IV, and V canals configuration in 49.9%, 9.3%, 2.2%, 32.6%, and 4%, respectively [5]. Anatomically, three-rooted maxillary premolars look similar to maxillary molars, and are sometimes called small molars or radiculous [6]. However, to the best of our knowledge, no case report of a maxillary second premolar with four roots has been published. We present here, a case report of a maxillary premolar with four roots: one mesiobuccal, one distobuccal, one mesiopalatal, and one distopalatal. The four roots were identified using cone-beam computed tomography (CBCT). A 45-year-old female patient with a no significant medical history was referred from a prosthodontist for endodontic treatment with the chief complaint of pain in her maxillary right second premolar. There was no swelling, sinus tract, or lymphadenopathy on the extraoral examination. The intraoral examination revealed no sinus tract or swelling.

**Figure 1:**
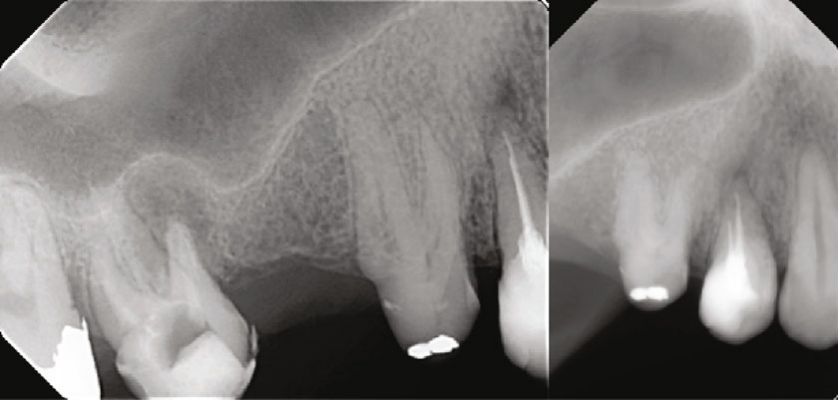
Periapical radiograph showing maxillary second premolar with abnormal morphology.

**Figure 2:**
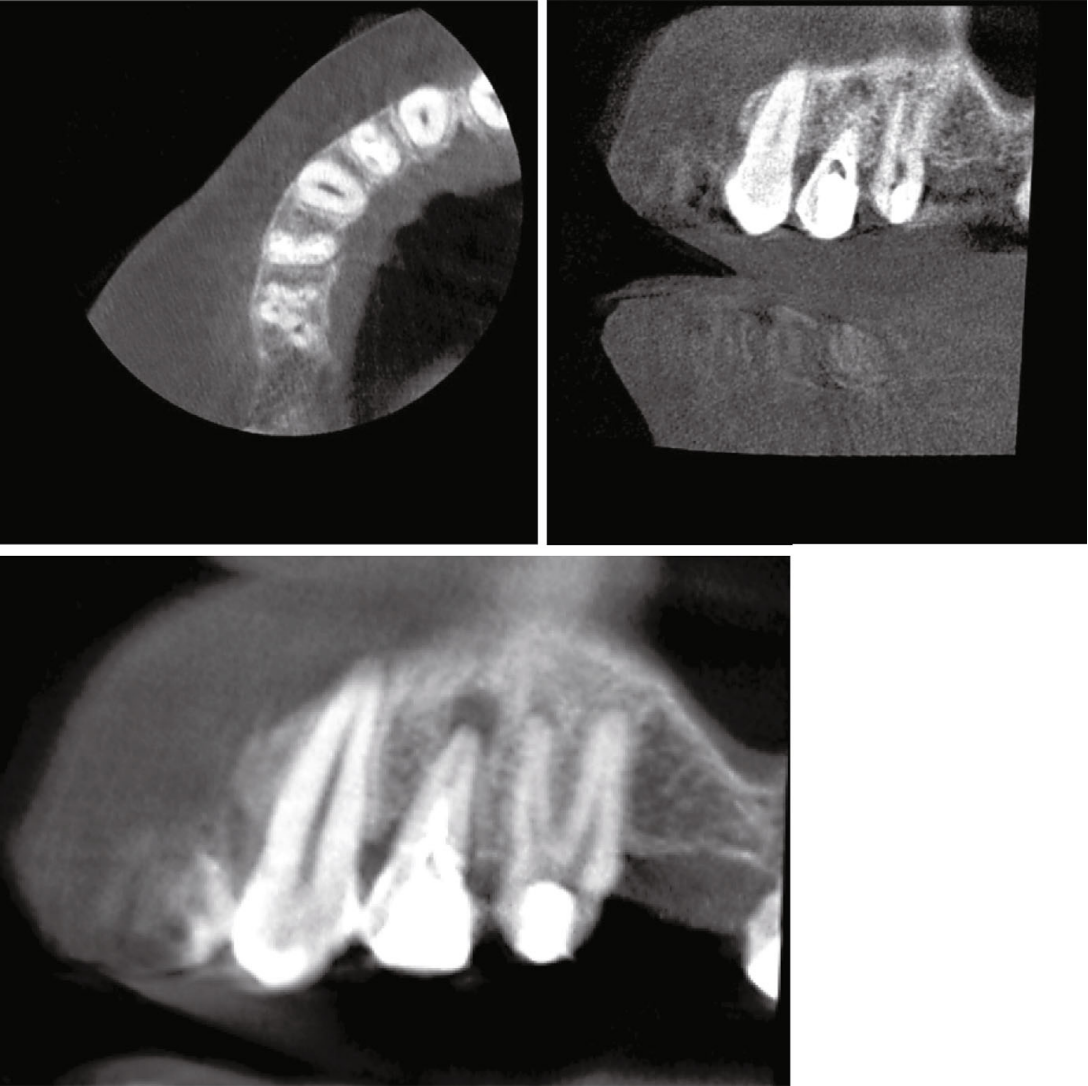
CBCT showing maxillary second premolar with 4 roots.

The tooth responded positively to the cold test (sensitive and lingering) with Endo-Frost (propane-butane mixture; Roeko, Germany) and was positive to percussion. The maxillary second premolar was diagnosed with symptomatic irreversible pulpitis and symptomatic apical periodontitis. Nonsurgical root canal treatment was recommended. Since the periapical radiograph revealed an unusual tooth anatomy and as there are limitations associated with the periapical radiograph, a cone-beam computed tomography (CBCT) was performed to confirm the number of roots and canals in the maxillary right second premolar. The CBCT confirmed the unusual anatomy and revealed four separate roots with four canals (Figures 1 and 2).

**Figure 3:**
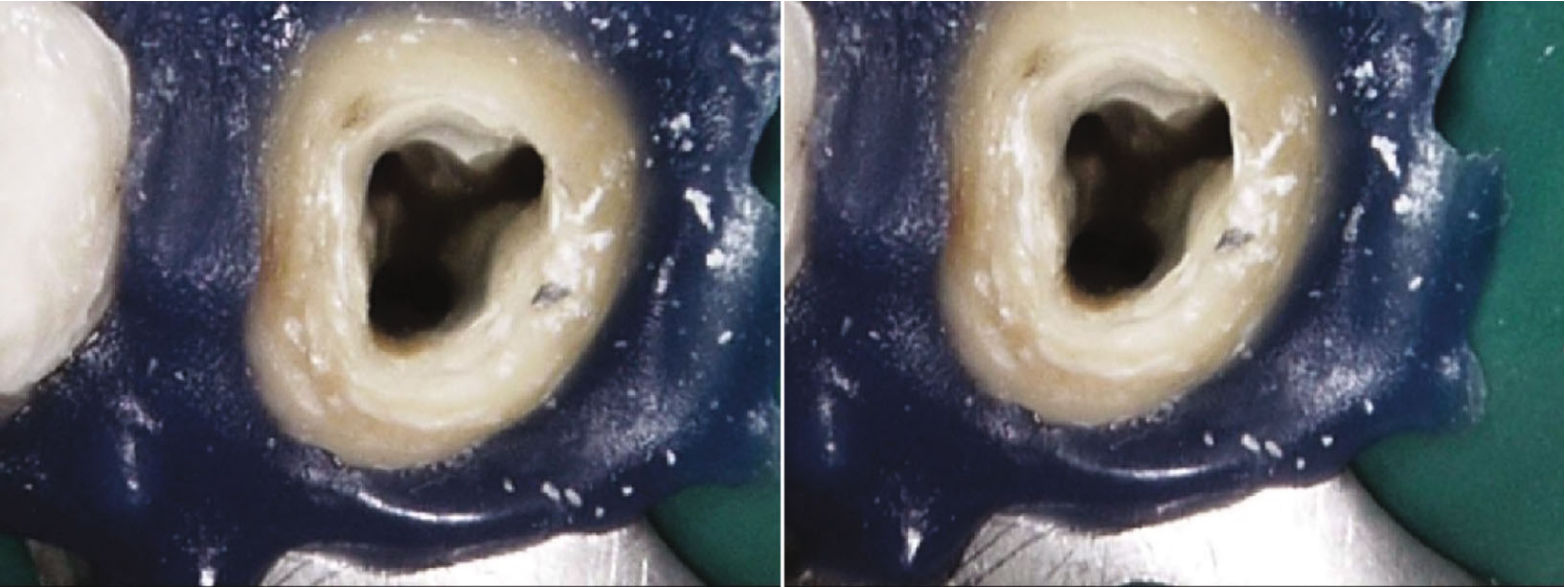
Access cavity photograph showing 4 canals orifices.

**Figure 4:**
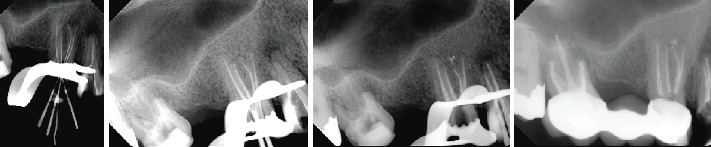
Radiographic taken during endodontic treatment, working length measurement, master cone, and final obturation after backfill soften root filling.

**Figure 5:**
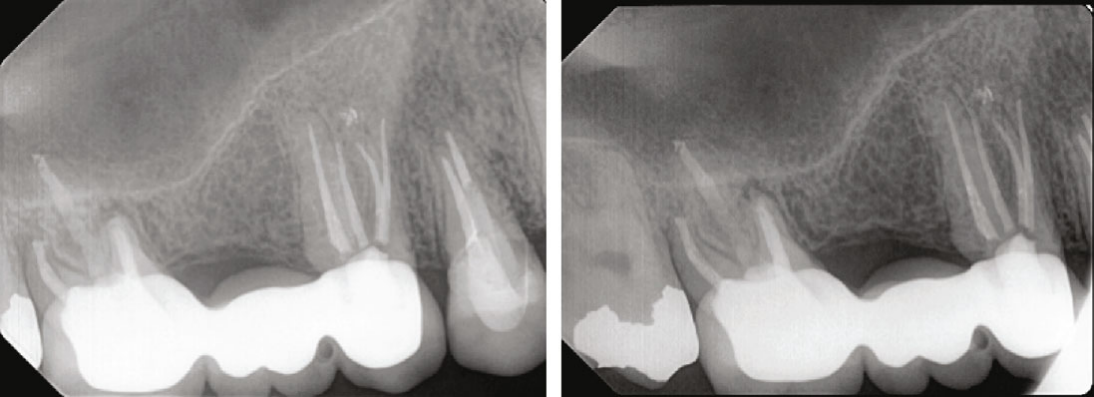
Two years follow up showing normal apical tissue.

Informed consent was obtained from the patient. Local anesthesia was administered (2% lidocaine with 1 : 100,000 epinephrine), and after rubber dam isolation, access preparation was performed. Inspection of the pulp floor under the dental operating microscope (Carl Zeiss, Germany) revealed four separate canal orifices (mesiobuccal, distobuccal, mesiopalatal, and distopalatal) (Figure 3). Initial orifice shaping was performed with an SX rotary file (Dentsply Tulsa, Tulsa, OK) starting with a K-file from size 8 and progressing up to size 15 (Dentsply Maillefer, Ballaigues, Switzerland). The root canal length was recorded using an apex locator, RootZX II (J. Morita, Tokyo, Japan), and confirmed with a radiograph. The canals were cleaned and shaped with nickel-titanium rotary instruments (ProTaper Next; Dentsply Tulsa). The canals were enlarged with Protaper X2 to the full working length. All the canals were irrigated with 5.25% sodium hypochlorite after each file, and a final rinse was performed with 17% ethylenediaminetetraacetic acid (EDTA) followed by 5.25% sodium hypochlorite. The canals were dried with sterile paper points (Dentsply Maillefer), and obturation was performed using the continuous wave technique. Four matching gutta-percha cones (Dentsply Maillefer) were gauged and carefully fitted with AH plus sealer (Dentsply Maillefer) under an operating microscope to working length. The downpack that was done with a System B Analytics unit (SybronEndo, Orange Country, CA) was used at a setting of 220°C for each canal. Backfilling was done using Obtura ІІІ (Max System, Obtura Spartan, Algonquin, IL, U.S.A). Finally, the access cavity was temporarily filled with Fuji resin-modified glass ionomer filling (ChemFil, Dentsply DeTrey, Germany). The patient was referred back to the prosthodontist for the final restoration (Figure 4). 2 years follow up clinical examination showing healthy gingiva with probing depth within normal limit, radiographic showing normal apical tissue (Figure 5).

## 2. Discussion

Adequate root canal therapy requires locating, cleaning, shaping, and obturating all the root canals. Failure of any of these steps can lead to posttreatment disease, pain, and/or complications in the treated tooth [1, 7]. Proper interpretation of conventional periapical radiographs taken in more than one angle is mandatory to detect any morphological variations in the teeth [8]. In addition, using advanced diagnostic radiographic techniques, such as CBCT, is very helpful in detecting such variations if conventional radiographic techniques do not provide adequate information and more details are required [9–12]. CBCT was performed in this case to aid the detection of morphological variations and for management. Furthermore, enhancing visualization by means of an operating dental microscope can aid proper examination of the floor of the pulp chamber, help localize canal orifices, and detect variations that may not be seen easily due to the limited access opening [13–15].

## 3. Conclusion

The clinical significance is that the second premolar with 4 roots is really a rare case.

## Data Availability

The data that support the findings of this study are available from the corresponding author upon reasonable request.
